# Antimicrobial Activities of Hexane Extract and Decussatin from Stembark Extract of *Ficus congensis*

**DOI:** 10.3390/ijms12042750

**Published:** 2011-04-21

**Authors:** Chinwendum Stephenie Alaribe, Francis Shode, Herbert A. B. Coker, Gloria Ayoola, Adesegun Sunday, Nisha Singh, Silva Iwuanyanwu

**Affiliations:** 1 Department of Pharmaceutical Chemistry, Faculty of Pharmacy, University of Lagos, Lagos, 234, Nigeria; E-Mails: habcoker@yahoo.com (H.A.B.C.); oyetayo68@yahoo.com (G.A.); 2 School of Chemistry, University of Kwazulu Natal, Westvile, P/Mail Bag X54001, Durban, 4000, South Africa; E-Mail: shodef@ukzn.ac.za; 3 Department of Pharmacognosy, Faculty of Pharmacy, University of Lagos, Lagos, 234, Nigeria; E-Mail: asegun67@yahoo.com; 4 School of Biological Studies, University of Kwazulu Natal, Westvile, P/Mail Bag X54001, Durban, 4000, South Africa; E-Mail: siveshnig@gmail.com; 5 Department of Mycology, College of Medicine, Lagos University Teaching Hospital, Lagos, 234 Nigeria; E-Mail: Sylva20042001@yahoo.com

**Keywords:** Moraceae, *Ficus congensis*, decussatin, antimicrobial, DAD, MHB

## Abstract

*Ficus congensis* (Moraceae) is used traditionally in the treatment of various diseases including infectious diseases, infertility, and gastrointestinal disorders. Investigation of hexane extract of the stem bark using chromatographic techniques led to isolation of a xanthone, 1-hydroxy-3,7,8-trimethoxyxanthone (Decussatin). The compound was elucidated based on spectroscopic methods such as nuclear magnetic resonance (NMR), UV, IR, and mass spectrometry (MS). Decussatin and the hexane extract were screened *in vitro* for antibacterial and antifungal activities using broth microdilution (MHB) and disc Agar diffusion (DAD) techniques against *Escheichia coli*, *Bacilus substilis*, *Klebsiela pneumonia*, *Staphylococcus aureus, Aspergillus fumigatus*, *Trichophyton mentagrophytes, Trichophyton rubrum* and *Candida albicans*. Hexane extracts showed potent antibacterial activity against *E. coli* and *B. subtilis* with minimum inhibitory concentrations (MIC) of 8 mg/mL and 5 mg/mL, respectively, while Decussatin of the highest concentration (8 mg/mL) used in this study showed no appreciable antimicrobial activity. Only hexane extract was active against *C. albicans* with a MIC of 1 mg/mL.

## 1. Introduction

The search for newer and effective antimicrobials is a continuous scientific exercise due to the resistance of microorganisms (bacteria, fungi, parasites, viruses) to currently existing drugs. Plants undoubtedly have been a veritable source of medicine to mankind. In drug discovery, biodiversity has been the main source of a host of ‘lead’ medicinal compounds for the discovery of effective and safe drugs. Ancient civilizations depended greatly on local flora and fauna for nutrition and health related purposes, experimenting with various berries, leaves, herbs and roots to find out what effect they had. As a result, some were successful in the management of different ailments such as fever, aches, infections, infertility and others.

*Ficus congensis* Engl (Moraceae) is a tropical tree up to 25 m high and of 2 m girth in trunk. It is a locally abundant terrestrial plant found in swamps and ravines and richly available in West Africa sub-region [1]. The genus *Ficus* is that of the fig trees and is centered in the tropics with about 755 species worldwide [2]. In the Afro-tropical region (Africa south of the Sahara including Madagascar), there are currently 112 recognized species; 36 of which are indigenous to southern Africa [3–6]. Many of them are used as food and for medicinal uses [7]. Studies have also shown that they are used for treatment of various diseases including infectious diseases, infertility, induction of labor and gastrointestinal disorders [8,9]. Anti-diarrheal, ulcer protective effects and acute toxicity of this plant had been investigated. Ethanol stembark extract demonstrated significant anti-diarrhea activity at 250 mg/mL (66.67% inhibition) and 500 mg/kg (88.89% inhibition). Oral administration of this extract produced a dose–dependent inhibition of ethanol induced gastric ulcer with maximal effect at 500 mg/kg (45.05%). The oral LD_50_ value obtained was >4,000 mg/kg in mice [10].

Nigeria is well endowed with fig tree species. The parts commonly used in Nigeria are the leaves, stem-bark, seed and root. The leaves are quite useful for preservation of kola nuts which are often used in the northern and eastern part of Nigeria. The leaves and stems are also used in southwest Nigeria to manage fever arising from infections. The bark is made into medication in northern Nigeria and is believed to promote physical fitness [1]. The aim of this present study is to investigate the anti-microbial activities of *F. congensis* with a view of isolating compound(s) responsible for these effects.

## 2. Results and Discussion

### 2.1. Chemical Constituents/Isolated Compound

Compound **1** was obtained as whitish yellow crystals exhibiting a M^+^ 302, corresponding to the molecular formula C_16_H_14_O_6_. The UV spectral data of compound **1** showed maximum absorption at λ_maxs_: 240, 260, 315 and 378 nm, which indicated the presence of tetraoxygenated moiety of the structure [1,3,7,8,11]. The ^1^H–NMR spectrum of **1** revealed three methoxys at δ 3.88 (3H, singlet), δ 3.81 (3H, singlet) and δ_H_ 3.76 (3H singlet) which are attached to the xanthone skeleton. A downfield singlet at δ_H_ 13.14 showed the presence of an unchelated hydroxyl group confirming hydroxyl substitution at position C-1 [12]. The presence of four aromatic protons at δ_H_ 6.22 (1H, doublet, J = 2.2 Hz, ArH), δ_H_ 6.20 (1H, doublet, J = 2.2 Hz, ArH), δ_H_ 7.22 (1H, doublet, J = 9.18 Hz, ArH) and δ_H_ 7.05 (1H, doublet, J = 9.18 Hz, ArH) indicated that compound **1** is a trisubstituted xanthone. The ^13^C NMR spectrum data also indicated the presence of 16 carbons with the carbonyl carbon appearing at δ 181.2.

**Figure 1.**
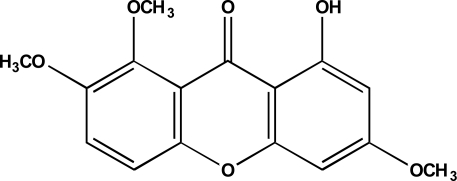
Structure of decussatin.

Analysis of the spectra data and comparison with literature [12] confirmed that compound **1** is 1- hydroxyl-3,7,8-trimethoxyxanthone, known as decussatin (see Figure 1).

Decussatin was previously isolated from a plant *Anthocleista vogelii* (Gentianaceae) [13] co-habiting with *F. congensis* (Moraceae) in the same swampy habitat suggesting that plants growing in the same habitat may have similar chemical constituents possibly through the same biochemical pathway and ecological route.

### 2.2. Structural and Spectra Information

#### Compound 1

1-Hydroxy-3,7,8-trimethoxyxanthone (Decussatin); Whitish-yellow crystals, C_16_H_14_O_6_; M·pt; 151–154 °C **IR v_max_ cm^−1^**: 2900, 2162, 1660, 1602, 1480, 1435, 1384, 1227, 1195, 1152.7, 1092, 1064, 981 for major peaks. **^1^H NMR** (400 MHz, CDCl_3_): δ 13.10 (1H, s, ArOH), 7.22 (1H, d, J 9.18 Hz), 7.05 (1H, d, J 9.18 Hz), 6.22 (1H, d, J 2.20 Hz), 6.20 (1H, d, J 2.20 Hz), 3.88 (3H, s, OMe), 3.81 (3H, s, OMe), 3.76 (3H, s, OMe); **^13^C NMR** (400 MHz, CDCl_3_): δ 181.1 (C-9), 166.4 (C-3), 163.9 (C-1), 157.1 (C-4a), 151.0 (C-5a), 149.3 (C-7), 148.9 (C-8), 120.5 (C-6), 115.8 (C-8a), 112.8 (C-5), 104.1 (C-9a), 96.8 (C-2), 92.0 (C-4), 61.8 (OCH_3_-8), 57.1(OCH_3_-7, 55.8 (OCH_3_-3).

### 2.3. Antimicrobial

The result of the MIC determination of hexane extract, decussatin and positive control neomycin against the four strains of bacteria tested is shown in Table 1. Hexane extract showed potent antibacterial activity against *E. coli* and *B. subtilis* with MIC of 8 mg/mL and 5 mg/mL, respectively, while Decussatin showed no significant antibacterial activity against any of the four tested bacteria. The antifungi studies on *F. congensis* showed that only hexane extract was active against *C. albicans* with MIC of 1 mg/mL while the activity of decussatin was not detected. They were both inactive against *A. fumigatus*, *T. mentagrophytes, and T. rubrum*.

Xanthones occupy an important position in the chemistry of natural products and have long been recognized to have choleretic, diuretic, antimicrobial, antiviral and cardiotonic action, and anticonvulsant activity, to mention but a few [14]. Recently, various bioactivities of xanthones including cytotoxic, antitumor, anti-inflammatory, enhancement of choline acetyltransferase activities and inhibition of lipid peroxidase have been described [15].

## 3. Experimental Section

### 3.1. Collection and Preparation of Plant Material

Stembark of *F. congensis* was collected in August 2007 from uncultivated land in University of Lagos, Akoka, identified and authenticated by M. O. Onadeji, Herbarium Officer at the Forest Research Institute of Nigeria (FRIN), Ibadan and voucher specimen (FHI NO 107844) was deposited at same institute. The stembark was air dried at 35 °C, pulverized using an electric grinder and kept in airtight containers at room temperature prior to use.

### 3.2. Extraction, Isolation and Structure Elucidation

The dried pulverized stembark of *F. congensis* (350 g) was extracted with hexane for 72 h at room temperature. The hexane fraction (18.50 g) was chromatographed on a column of silica gel (140 g) and eluted with hexane—EtOAC (10:1; 1–10) to give Fractions 1–19 of 100 mL each. The fractions thus obtained were compared using TLC (fluorescence silica gel plates using hex- EtOAC {8:2}) as solvent. Those giving similar spots were combined. Fractions 4–7 was further eluted with hex—EtOAC (8:2), evaporated and the white yellow precipitate obtained was recrystallized from methanol to afford Compound **1** which was successively subjected to ^1^H NMR, infra red (IR), UV, MS and ^13^C NMR analysis for structure determination.

The ^1^H-NMR and ^13^C-NMR were recorded in CDCl_3_ with TMS as internal standard on a Bruker AMX-400 Ultrashield NMR spectrometer operating at 400 and 75 MHz. The infrared (IR) was recorded on the Perkin Elmer-Universal ATR sampling Accessory, FT-IR spectrometer. The ultraviolet (UV) spectra were taken in DCM and recorded on Lambda 18 UV/VIS Spectrometer. Si gel refers to Merck Kieselgel 60 (70–230 and 230–400 mesh ASTM). All other reagents and chemicals used were of analytical grade.

### 3.3. Microdilution Bioassay

Microdilution bioassay method was carried using the microplate method of Eloff [16] with modifications. The hexane extract and decussatin were screened against ATCC culture strains of *Escherichia coli* (ATCC 11775), *Bacillus subtilis* (ATCC 6051), *Klebsiella pneumoniae* (ATCC 13883) and *Staphylococcus aureus* (ATCC 12600) obtained from The School of Biological and Conservation Sciences, University of Natal, Pietermaritzburg Campus, South Africa. The hexane extract (1–8 mg/mL, 100 μL) and decussatin (8 mg/mL) were introduced separately into 96 well microplates and 100 μL bacterial culture were added to each well. Neomycin (100 μg/mL) was used as positive control, 10% tween 80 used to dissolve the extract was used as negative control and bacterial free wells were used as blank controls. The microplates were covered with parafilm and incubated for 24 h at 37 °C. 40 μL of 0.2 mg/mL of p-iodonitrotetrazolium violet (INT) were added to the wells to serve as an indicator of bacterial growth and incubated at 37 °C for 30 min. The minimum inhibitory concentration (MIC) was taken as the lowest concentration of the extract or compound that completely inhibited bacterial growth. All determinations were in triplicates.

### 3.4. Antifungal Assay

Disk Agar Diffusion (DAD) method [17] was used in this study. The plates were prepared by pouring about 15 mL of molten Sabouraud dextrose agar into sterile petri dishes. The plates were allowed to solidify and 0.1% inoculums suspension of four clinically isolated fungi *A. fumigatus*, *T. mentagrophytes, T. rubrum* and *C. albicans* were separately swabbed uniformly and incubated at 25–30 °C. The cultures were examined every 2–3 days under the microscope for evidence of growth for 6 weeks. Different concentrations of hexane extract (1–5 mg/mL) and decussatin (10 mg/mL) were loaded on sterile individual discs. The discs were placed on the surface of the medium and the plates were incubated at 30 °C for 24 h. Nystatin (100 μg/mL) and clotrimazole (100 μg/mL) were used as positive controls. Negative controls were prepared using 10% tween 80 and distilled water. The zones of inhibition around the disc were measured in millimeters. All determinations were in triplicates.

## 4. Conclusions

In summary, the phytochemical studies on hexane stembark of *Ficus congensis* led to the isolation of decussatin, a tetraoxygenated xanthone. The hexane extract exhibited notable antimicrobial activity against *E. coli*, *B. subtilis* and *C. albicans.* Decussatin did not show appreciable antimicrobial activities within the tested concentrations isolated and used in this study.

## Figures and Tables

**Table 1.** Minimum Inhibitory Concentration (MIC) of Decussatin and Hexane extract against four bacteria.

**Test strain (bacteria)**	**MIC (**mg/mL**)**		
	**Hexane extract**	**Decussatin**	**Neomycin**
*E. coli*	8.00	N.D	0.10
*B. substilis*	5.00	N.D	1.56
*K. pneumonia*	N.D	N.D	1.20
*S. aureus*	N.D	N.D	0.39

* All MIC values in mg/mL with the exception of Neomycin in μg/mL; N.D: Not detected within the tested concentration (8 mg/mL).
